# Synergism between coexisting eye diseases and sex in increasing the prevalence of the dry eye syndrome

**DOI:** 10.1038/s41598-023-50871-1

**Published:** 2024-01-03

**Authors:** Andreas Stang, Börge Schmidt, Sara Schramm, Bernd Kowall, Karl-Heinz Jöckel, Raimund Erbel, Oliver Kuss, Gerd Geerling

**Affiliations:** 1 Institute of Medical Informatics, Biometry and Epidemiology, University Hospital Essen, Hufelandstr. 55, 45147 Essen, Germany; 2 School of Public Health, Department of Epidemiology, Boston University, Boston, USA; 3 Institute for Biometrics and Epidemiology, German Diabetes Center (DDZ), Leibniz Center for Diabetes Research at Heinrich Heine University, Auf’m Hennekamp 65, 40225 Düsseldorf, Germany; 4 Centre for Health and Society, Faculty of Medicine, Heinrich Heine University Düsseldorf, Düsseldorf, Germany; 5 Department of Ophthalmology, University Hospital Düsseldorf, Moorenstraße 5, 40225 Düsseldorf, Germany

**Keywords:** Diseases, Medical research, Risk factors

## Abstract

The aim was to investigate prevalence of dry eye syndrome (DES) in a population-based sample in Germany. The association between coexisting eye diseases and DES was also of interest. We recontacted participants of the Heinz Nixdorf Recall study between 2018 and 2021 by postal questionnaire that included the Women’s Health Study questionnaire on DES. We estimated prevalence of DES and examined DES-associated factors among 2095 participants aged 62–91 years. We performed interaction analyses between sex and coexisting eye diseases in relation to the DES prevalence and performed bias analyses to examine the robustness of the results. The DES prevalence was 31.5% (34–36% after correction for potential non-response bias, 24.1% after correction for outcome misclassification) and it was almost 2.1-times higher in women than in men (women 42.3%, men 20.4%). Among DES subjects, 70.3% had received treatment in the previous 12 months. There was synergism between female sex and coexisting eye diseases (cataract, glaucoma, macular degeneration) in terms of DES prevalence. The extrapolated numbers of patients aged 62–91 years with DES in Germany are 1.1–1.3 million men and 6.1–6.8 million women. The observed synergism may be explained by differences in ocular physiology, subjective perception and response behavior. Women with eye diseases (cataract, glaucoma, macula degeneration) appear to have a markedly higher susceptibility to suffer from DES than men, so that a diagnostic workup of DES symptoms is particularly justified in women with these eye diseases.

## Introduction

Dry eye syndrome (DES) is a form of keratoconjunctivitis that causes a variety of symptoms including dryness, irritation, itching, burning and fluctuating vision as it affects the tear film and ocular surface. DES is associated with reduced vision-related quality of life^1^. It can be diagnosed by a variety of clinical tests including the Schirmer test, tear break-up time and vital staining of the ocular surface^2^. Based on the Physicians' Health Study and the Women's Health Study, it was estimated that at an age of 50 years and older 4.4% of all men (1.68 million) and 7.8% of all women (3.23 million) in the United States are affected^3,4^.

The review of the epidemiology of DES by the Tear Film and Ocular Surface Society (TFOS) revealed that the prevalence depends on sex (higher prevalences among women) and age (higher prevalences among the elderly)^5^. The Dutch population-based Lifelines cohort study including 79,866 participants showed that the DES prevalence as based on data of the first follow-up 2014–2017 is 3–5% and 8–13% within age groups below age 60 years among men and women, respectively so that DES is not just a problem for older people. Among the many factors studied in Lifelines, macular degeneration, glaucoma or ocular hypertension, allergic conjunctivitis, keratoconus, and cataract surgery were associated with an increased prevalence of DES^6^. There are only a few DES incidence studies. The U.S. Beaver Dam Eye Study that started baseline assessment in 1993 estimated a DES incidence of 13.3% over five years among Caucasians aged 48–91 years. The age-adjusted DES incidence was higher among women (25%) than men (17%) over a 10-year period^7^. The Twins UK Study estimated a DES incidence as defined by Women’s Health Study (WHS) criteria of 4.4% in females aged 20–87 years in a 2-year period^8^. There are several treatments for DES including artificial tears, anti-inflammatory medication and treatment of associated eyelid diseases.

The only available estimates of the prevalence of DES in Germany come from an analysis of claims data from a single Statutory Health Insurance company, which included approximately 3.6 million insured persons aged 18 years and older between 2008 and 2015. The estimated prevalence in 2014 was 2.3%^9^. The aim of this study was to investigate the age- and sex-specific prevalence of DES in a population-based sample in north-western Germany. The association between pre-existing eye diseases and DES was also of interest.

## Results

The mean age of the 2095 respondents was 74 years, ranging from 62 to 91 years. From the age of 87, the absolute number of study participants is small (Suppl. Fig. S1, Appendix). Overall, 31.5% had a DES. The prevalence of DES was almost 2.1-times higher in women than in men (men 20.4%, women 42.2%). Overall, 70.3% of the 625 subjects reported having been treated for DES in the previous 12 months (men 60.6%, women 74.9%). Regardless of the presence of DES, women were more likely to report feeling dry eyes and eye irritation. By far the most common self-reported eye condition confirmed by a physician was cataract (42.2%), followed by glaucoma (8.5%) and macular degeneration (5.4%). Women had a slightly higher prevalence of coexisting eye disease than men. Among the 625 subjects with DES, treatment by an ophthalmologist (with or without self-therapy) in the 12 months prior to the survey was the most frequently reported form of treatment (total 46.4%, men: 41.0%, women 49.0%). Men stated more frequently that they had not had any treatment in the last 12 months. Self-treatment alone took place in 17.8% of cases (men: 15.2%, women: 19.1%) (Table 1).

**Table 1:** Dry eyes syndrome and coexisting eye diseases among 2095 men and women of the Heinz Nixdorf Recall study, Germany, January 2018–September 2021.

Characteristic	Overall		Men		Women	
Number of subjects, n %	2095	100	1034	49.4	1061	50.6
Age (years) mean (SD)	74.0	6.9	74.2	6.9	73.8	7.0
Age groups (years) n, %						
62–66	364	17.4	169	16.3	195	18.4
67–71	514	24.5	253	24.5	261	24.6
72–76	427	20.4	209	20.2	218	20.6
77–81	447	21.3	231	22.3	216	20.4
82–86	253	12.1	129	12.5	124	11.7
87–91	90	4.3	43	4.2	47	4.4
Physician-confirmed dry eyes n, %						
No	1420	70.5	809	81.1	611	60.0
Yes	595	29.5	188	18.9	407	40.0
Missing	80		37		43	
Frequency of feeling dry eyes n, %						
Constantly	71	3.6	22	2.2	49	4.9
Often	245	12.4	70	7.1	175	17.5
Sometimes	763	38.5	351	35.7	412	41.2
Never	904	45.6	540	54.9	364	36.4
Missing	112		51		61	
Frequency of feeling eye irritation n, %						
Constantly	35	1.7	14	1.4	21	2.1
Often	214	10.6	73	7.4	141	13.8
Sometimes	1083	53.7	520	52.4	563	55.1
Never	683	33.9	386	38.9	297	29.1
Missing	80		41		39	
Dry eye syndrome						
No	1361	68.5	780	79.6	581	57.7
Yes	625	31.5	200	20.4	425	42.3
Missing	109		54		55	
Treatment in the recent 12 months among subjects with dry eye syndrome (n = 625)						
No treatment	182	29.7	78	39.4	104	25.1
Ophthalmologist (MD)	234	38.2	70	35.4	164	39.6
Ophthalmologist (MD) and self treatment	50	8.2	11	5.6	39	9.4
MD of another speciality	2	0.3	1	0.5	1	0.2
Pharmacist	15	2.5	3	1.5	12	2.9
Pharmacist and MD of another speciality	11	1.8	3	1.5	8	1.9
Self treatment	109	17.8	30	15.2	79	19.1
Self treatment and pharmacist	9	1.5	2	1.0	7	1.7
Missing	13		2		11	
Self-reported coexisting cataract						
No	1179	57.8	613	60.5	566	55.1
Yes	861	42.2	400	39.5	461	44.9
Missing	55		21		34	
Self-reported coexisting glaucoma						
No	1826	91.5	917	92.3	909	90.6
Yes	170	8.5	76	7.7	94	9.4
Missing	99		41		58	
Self-reported coexisting macula degeneration						
No	1898	94.6	942	95.2	956	94.0
Yes	109	5.4	48	4.9	61	6.0
Missing	88		44		44	

**Figure 1:**
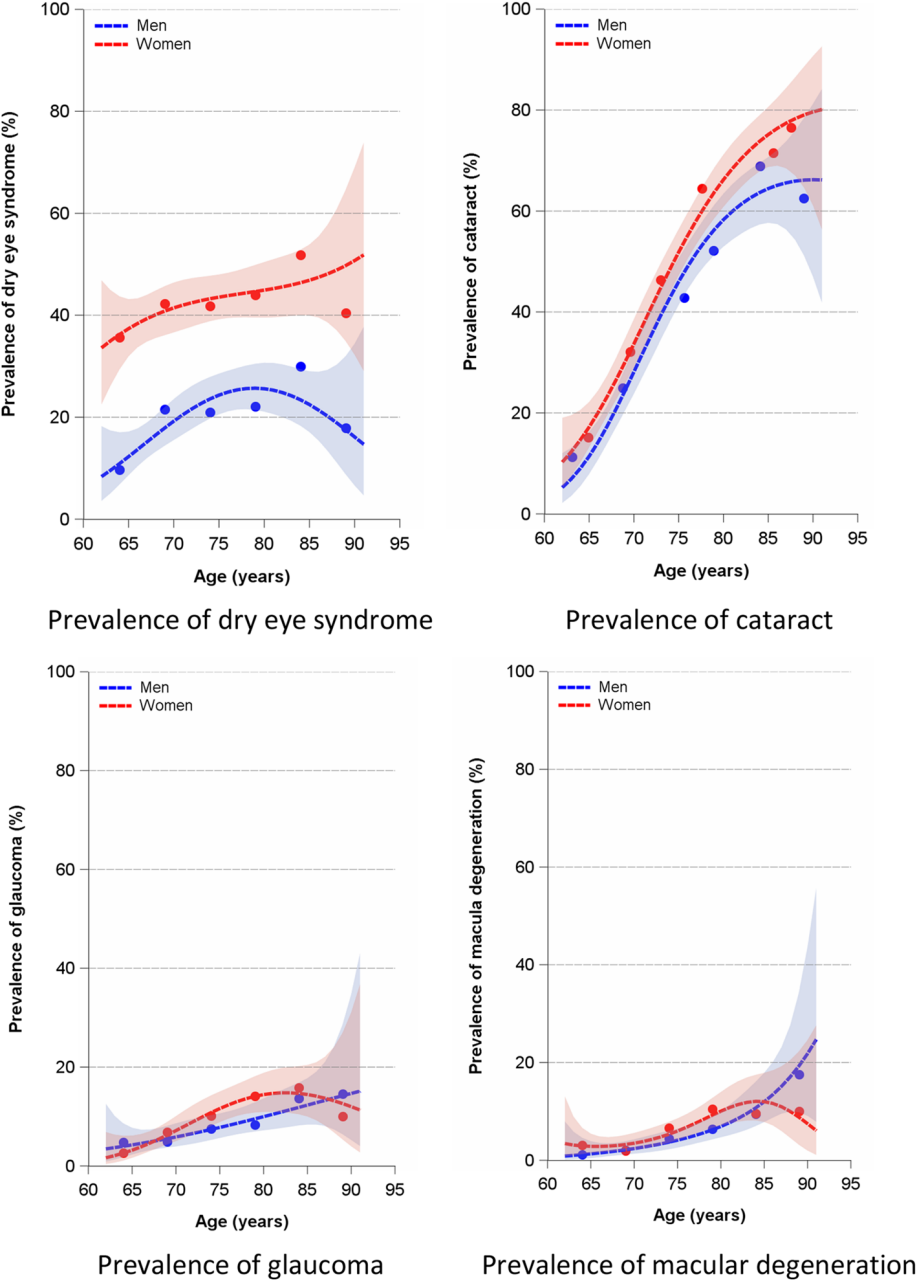
Prevalence of dry eye syndrome, cataract, glaucoma, and macular degeneration by sex and age among 2095 men and women of the Heinz Nixdorf Recall study, Germany, January 2018–September 2021. Points indicate prevalence by midpoints of the 5-year age groups; flexible logistic regression modeling included age, age^2^, and age^3^.

The age-specific prevalence of DES and coexisting eye diseases increases markedly with age. The greatest increase in age-specific prevalence is seen for cataract, where the prevalence is well over 50% in the highest age groups. Due to the small number of subjects in the age group 87 years and older, a reliable estimate of the prevalence in this age group is hardly possible. The results of the flexible modelling also indicate this by the large width of the confidence intervals (Fig. 1). The extrapolated number of patients with DES in Germany based on age- and sex-specific multiple bias-corrected prevalences results in a number of 1.1–1.3 million men and 6.1–6.7 million women with DES aged 62–91 years.

The prevalence of DES was higher in the presence of coexisting eye disease. The prevalence of DES differed most with the presence of cataract (age-adjusted prevalence difference for DES 17.3% points). Age-adjustment had little effect (Table 2). When we studied the joint effect of sex and coexisting eye disease (cataract, glaucoma, macular degeneration separately) on the DES prevalence, we found different magnitudes of synergism. The example of cataracts illustrates this: men with cataract have an 11.2% points higher DES prevalence than men without cataract. Women without cataract have a 16.9% points higher DES prevalence than men without cataract. If the effect of both exposures (cataract and female sex) occurred at the same time in a purely additive manner, one would expect a 11.2% points + 16.9% points = 28.1% points higher prevalence than in men without cataract, i.e. 16.1% points + 28.1% points = 44.2% points. In fact, the prevalence in women with cataract was 53.4%, an excess of 9.2% points. Synergism between female sex and eye diseases accounted for 17.7%, 19.3%, and 25.3% of the DES prevalence among women with cataract, glaucoma, and macular degeneration, respectively (Table 3).

**Table 2:** Association between the presence of coexisting eye diseases and dry eye syndrome among 2095 men and women of the Heinz Nixdorf Recall study, Germany, January 2018–September 2021.

	Dry eyes	N	DES Prev (%)	Crude PD	95%CI	Adj. PD	95%CI
Cataract							
No	No	862		17.1	12.9; 21.3	17.3	12.8; 21.9
	Yes	275	24.2				
Yes	No	482					
	Yes	339	41.3				
Glaucoma							
No	No	1233		13.9	6.0; 21.8	12.6	4.6; 20.6
	Yes	528	30.0				
Yes	No	92					
	Yes	72	43.9				
Macular degeneration							
No	No	1273		5.0	− 4.6; 14.6	3.5	− 6.1; 13.2
	Yes	562	30.6				
Yes	No	65					
	Yes	36	35.6				

*Prev* prevalence of DES, *crude PD* crude prevalence difference of DES in relation to the presence or absence of eye diseases, *adj. PD* age-adjusted prevalence difference, *95%CI* 95% confidence interval; missing data excluded.

**Table 3:** Positive interaction between sex and coexisting eye diseases in relation to the prevalence of dry eye syndrome among 2095 men and women of the Heinz Nixdorf Recall study, Germany, January 2018–September 2021.

Sex	Coexisting eye disease	N	Dry eyes (%)	Stratified prevalence differences (%)	IC (%) and 95%CI	Attributable proportion due to interaction (%) and 95% CI
Cataract						
Male	No	591	16.1	0	9.2 (1.1; 17.4)	17.7 (3.3; 32.0)
	Yes	381	27.3	11.2		
Female	No	546	33.0	0		
	Yes	440	53.4	20.4		
Glaucoma						
Male	No	881	19.8	0	11.3 (− 3.6; 26.2)	19.3 (0.8; 37.9)
	Yes	75	26.7	6.9		
Female	No	880	40.2	0		
	Yes	89	58.4	18.2		
Macular degeneration						
Male	No	913	20.0	0	12.6 (− 4.8; 30.1)	25.3 (− 5.1; 55.7)
	Yes	43	16.3	− 3.7		
Female	No	922	41.1	0		
	Yes	58	50.0	8.9		

*IC* interaction contrast, that is, difference of prevalence differences; missing data excluded; age-adjusted IC for cataract 9.0% (0.9; 17.2), for glaucoma 11.7% (− 3.3; 26.7), and for macular degeneration 14.5% (− 3.0; 31.9); age-adjusted attributable proportions due to interactions for cataract 17–2 (2.7; 31.6), for glaucoma 17.4 (− 6.3; 41.0), and for macular degeneration 24.4 (− 5.7; 54.5).

Our quantitative non-response bias analysis of four hypothetical non-response bias scenarios shows that the bias corrected overall prevalence of DES ranges between 35.4% and 39.0% (men: 22.7–25.0%, women: 46.5–51.4%) (Suppl. Table S3, Appendix). For men, the bias analyses show that the responder-based and bias-corrected (responder plus nonresponder) prevalence were very similar up to age 70, thereafter the bias-corrected prevalence was higher than the responder-based prevalence. The decline in prevalence among older women (analysis of responders only) is no longer evident when correcting for non-response bias. (Suppl. Fig. S2, Appendix). Our quantitative bias analysis for outcome misclassification shows that with a specificity and sensitivity of 83% and 77%, respectively, as published by Gulati et al.^2^, the true prevalence of DES would be 24.1% instead of 31.5%.

## Discussion

In this population-based study of 2095 men and women aged 62–91 years, we found a DES prevalence of 31.5%. Among the DES subjects, 70.3% had received treatment in the previous 12 months. Specifically, subjects with cataract and glaucoma had a higher DES prevalence than subjects without these eye diseases. There was a synergism (positive interaction) between female sex and coexisting eye disease in terms of DES prevalence. In simple terms, there are cases of DES that only occurred because the coexisting eye disease was in women. These extra cases would not have been observed in men. Various bias analyses have provided insight into the robustness of the study results.

Both the TFOS DEWS II (dry eye workshop) epidemiology report and a recent systematic review showed that the definition of DES (self-report, clinical examination) varies widely between studies. In addition, study design characteristics (age range, sex, population-based versus patient-based surveys) contribute to this variation^5,10^. However, there is consistent evidence that the prevalence of DES increases markedly with age, particularly from around 50 years of age, and that women have a higher prevalence of DES than men in every age group^3–6,9,11^. The higher prevalence of DES in women may be explained by higher rates of diseases and/or their treatment associated with DES (e.g. autoimmune diseases such as Sjögren's syndrome, rheumatoid arthritis, systemic lupus erythematosus, thyroid diseases, migraine, depression)^12^ and hormone replacement therapy^13,14^. Sex differences in ocular physiology of the meibomian glands, lacrimal glands, conjunctiva and cornea are also thought to play a role^12^.

We have observed that the prevalence of DES is higher in the presence of coexisting eye diseases (cataract, glaucoma or macular degeneration) than in the absence of such diseases. Siffel et al. found that cataract (48.5%), glaucoma/ocular hypertension (19.5%) and macular degeneration (13.0%) were the most common diseases present before a new diagnosis of DES. Cataract surgery was the most common procedure prior to the occurrence of DES^9^. Erb et al. found that the incidence of DES increased with the use of three or more antiglaucoma medications and increased with the duration of glaucoma^15^. The Lifelines Cohort Study showed a higher prevalence of DES in people with macular degeneration, glaucoma/ocular hypertension, cataract surgery, glaucoma surgery and other conditions^6^.

The results of our interaction analyses suggest that there is public health relevant synergism (positive interaction) between female sex and eye diseases and/or their related treatments in relation to the prevalence of DES. There are several possible explanations for this synergism. On the one hand, subjective perception and response behaviour may differ between men and women in general and with DES. In a detailed survey of study participants in the Physicians' Health Study and the Women's Health Study who were classified as DES + on the basis of the WHS questionnaire, it was shown that women's self-reported eye complaints due to DES were more severe and that women used therapies more frequently than men. The authors suggested that this sex difference may be explained by a more intense perception of pain in women, which has also been shown in experiments^16^. On the other hand, sex differences in ocular physiology and the sex hormone dependence of DES could also be an explanation. The results of our interaction analyses imply that in the case of eye diseases (cataract, glaucoma, macular degneration) and/or their related treatments, women have a markedly higher susceptibility to suffer from DES than men, so that a diagnostic workup of DES symptoms is particularly justified in women with these eye diseases. Furthermore, pathogenetic studies that take into account the particularities of female ocular physiology and hormonal status could provide further mechanistic insights into the etiology of DES.

Strengths of our study are the study size and the good epidemiologic characterization of the study participants, who were drawn from a population-based study. We also captured DES treatment need in our study. However, our study has limitations. First, there was a relevant proportion of people who did not take part in the baseline examination in 2000–2003. Furthermore, there was also non-response in our eye survey years after baseline assessment. Our analysis of potential non-response bias showed that the non-response bias corrected prevalence estimate for DES increased from 31.5 to 35.4–39.0%, depending on the assumed bias scenario. Second, we did not validate self-report of clinician-diagnosed DES. Using diagnostic indices from a small study that validated the WHS questionnaire against standardized ophthalmologic examination in 53 subjects^2^, the bias analysis showed that the prevalence of DES is overestimated (uncorrected 31.5%, bias-corrected 24.1%). Third, the proportion of very old subjects (87 years and older) was quite small, so that the age-prevalence modelling was quite imprecise in these old people.

## Conclusions

DES has a high prevalence in the elderly in Germany and coexisting eye diseases are associated with a higher prevalence of DES. The observed synergism may be explained by differences in ocular physiology and by differences in subjective perception and response behavior.

## Material and methods

The rationale, design, and methods of the Heinz Nixdorf Recall study have been described in detail^17,18^. In brief, between December 2000 and August 2003, we recruited 4,814 men and women aged 45–75 years residing in the industrial cities of Essen, Bochum, and Mülheim in the Ruhr area of Germany from mandatory residence lists, with a response proportion of 56%^19^. Primary end points for this study were based on unequivocally documented coronary events that met predefined study criteria.

Annual postal questionnaires assessed the morbidity status (i.e., medication, hospital admissions, outpatient diagnoses of cardiovascular disease) during follow-up. Self-reported incident cardiovascular morbidity was validated by review of hospital and physician records. All death certificates in the three study cities were screened regularly. Deceased participants were traced back in time to obtain as much information as possible to verify the cause of death.

The recruitment results at baseline (2000–2003) and in the follow-up (2018–2021), in which the DES survey took place, including a presentation of non-responders, are shown in Supplementary Fig. S3. Our postal questionnaire that included questions related to dry eye syndrome (see questionnaire in the Supplementary File S1) was sent between January 6, 2018 and September 16, 2021. Overall 2095 subjects participated.

We used the 3-item questionnaire of the Women’s Health Study (WHS)^4^. Question #1 asked whether subjects had ever been diagnosed with dry eye syndrome by a clinician. Question #2 asked about the frequency of feeling dry eyes and question #3 asked about the frequency of irritation. The WHS questionnaire was validated against a detailed questionnaire containing 19 questions about dry eye. The estimated specificity and sensitivity were 94% and 60%, respectively^20^. Another study validated the WHS questionnaire against clinical tests among 53 subjects, with an estimated specificity and sensitivity of 83% and 77%, respectively^2^. The WHS questionnaire has been used in several surveys on DES^3,4,6,13,21^.

The WHS questionnaire does not provide any information about the patient’s previous treatment history. However, self-treatment and treatment based on non-ophthalmologist recommendation is frequent and may delay appropriate provision of care. For this reason, we also assessed the patient’s treatment history in more detail and included additional questions that addressed treatment history. Subjects were also asked if a clinician had ever diagnosed them with cataract, glaucoma, or macular degeneration.

The English-language WHS questionnaire was translated using a forward and backward approach. AS and GG first translated the English-language version of the questionnaire into German. Subsequently, a certified translator then translated the German version back into English without knowing the original English version. Minor discrepancies between the back-translated version and the original English-language version were clarified and agreed with the translator.

The study was approved by the Ethics Committee of the Medical Faculty of the University of Duisburg-Essen (AZ 99-69-1200, 12 May 1999). Written informed consent was obtained from all subjects. The study was certified and re-certified according to DIN EN ISO 9001:2000 by TÜV Rheinland.

We applied the definition of DES as it has been repeatedly applied in the past based on the WHS questionnaire. DES is defined as a self-reported diagnosis of DES by a physician and/or self-reported severe symptoms (dryness and irritation either constantly or often)^13^. We estimated the age- (62–66, 67–71,…, 87–91 years) and sex-specific prevalence of DES and eye diseases. We used logistic regression with age, age^2^ and age^3^ as the independent variables to flexibly model the association between age and DES or eye diseases. Furthermore, we used linear regression to estimate crude and age-adjusted prevalence differences and 95% confidence intervals (95% CI) of DES in relation to the coexisting eye diseases^22^.

We were interested in the public health relevance of potential interaction between sex and coexisting eye diseases which requires the assessment on an additive scale^23^. We therefore studied the joint effect of sex (women counted as exposed) and coexisting eye disease (cataract, glaucoma, macular degeneration) on the DES prevalence on the additive scale. For this, we formed four subgroups: (1) male subjects without coexisting eye disease (double unexposed, P_00_), (2) male subjects with coexisting eye disease (P_01_), (3) female subjects without coexisting eye disease (P_10_) and (4) female subjects with coexisting eye disease (double exposed) (P_11_). We estimated the difference of prevalence differences [P_11_–P_10_] − [P_01_–P_00_] which is called interaction contrast (IC). Given that both exposures are associated with an increased prevalence of DES, an IC > 0 indicates synergism (so called super-additivity) and means that the joint effect of female sex and coexisting eye disease is larger than the effect expected from the sum of the individual effects. Furthermore, we calculated the attributable proportion due to interaction which expresses the proportion of the DES prevalence among women with coexisting eye diseases (that is among those doubly exposed) that is due to synergistic interaction, that is IC/P_11_^24^.

We performed quantitative bias analyses to examine the effect of non-response on the prevalence and age pattern of DES^25^. The distribution of responders and non-responders (baseline non-responders and non-responders at follow-up) by sex and age is presented in Supplementary Table S1. For non-participants, we used the midpoint of the time interval of the receipt of the questionnaires as the date for calculating age. It has been repeatedly shown that non-response in baseline recruitment as well as non-response in the follow-up of cohort studies is associated with poorer health status^26–28^. We therefore assumed two non-differential non-response scenarios where the DES prevalence of non-responders is a constant percentage (above 100%) of the DES prevalence of responders (scenario #1 and #2) and two non-response scenarios in which this percentage increases with age among the nonresponders (scenario #3 and #4) (Suppl. Table S2). We compared the DES age prevalence pattern of responders with the DES age prevalence pattern of the total population (responders and non-responders) by re-running the flexible logistic regression models as described above.

In another bias analysis, we used the diagnostic indices (sensitivity and specificity) obtained from the validation studies to estimate the true prevalence of DES. If A* is the number of participants classified with DES, N is the total number of individuals, Se is the sensitivity, Sp is the specificity and Fp is the false positive proportion, the number of true DES subjects is calculated as (A*-Fp*N)/(Se + Sp-1)^25^.

To estimate the number of prevalent cases in Germany, the non-response-corrected prevalences were first determined for each age and sex group as part of the bias analyses (4 non-response scenarios). Subsequently, the outcome misclassification was corrected in each stratum using the data from Gulati (specificity 0.83, sensitivity 0.77)^2^. The prevalence corrected for both bias sources was then used to estimate the nationwide number of prevalent DES cases for the age range 62–91 years using the population figures for Germany as of December 31, 2021. We report the minimum and maximum estimated number of DES cases per sex to illustrate the uncertainty of the bias analyses. We have assumed that the bias-corrected prevalence estimates from this study are representative for Germany.

We calculated and report confidence intervals to assess the precision of our estimates because our goal is estimation and not significance testing. We wish to avoid publication bias by preferential reporting of significant results. Instead, we judge the value of our estimates by their precision and validity^29,30^. All statistical analyses were done with SAS 9.4 (SAS Institute Inc., Cary, North Carolina).

### Ethics approval

The study was approved by the Ethics Committee of the Medical Faculty of the University of Duisburg-Essen (AZ 99-69-1200, 12 May 1999).

### Consent to participate

Informed consent was obtained from all individual participants included in the study.

### Supplementary Information

Supplementary Information 1.

Supplementary Information 2.

## Data Availability

Due to the sensitive nature of the questions asked in this study, survey respondents were assured raw data would remain confidential and would not be shared.
