# Supplementary material for: Synergism between coexisting eye diseases and sex in increasing the prevalence of the dry eye syndrome
Source: Sci Rep. 2024 Jan 3;14:314. doi: 10.1038/s41598-023-50871-1 (PMC10764946; doi:10.1038/s41598-023-50871-1)
Supplement: Supplementary file 1 — Supplementary Information 1. [file 41598_2023_50871_MOESM1_ESM.docx]

**S U P P L E M E N T**

**Synergism between coexisting eye diseases and sex in increasing the prevalence of the dry eye syndrome**

Andreas Stang*,^1-2^ Börge Schmidt,^1^ Sara Schramm,^1^ Bernd Kowall,^1^ Karl-Heinz Jöckel,^1^ Raimund Erbel,^1^ Oliver Kuss,^3-4^ Gerd Geerling,^5^

**Suppl. Table 1 Sex- and age distribution of responders and non-responders at baseline and nonresponders at follow-up, Heinz Nixdorf Recall study, Germany, January 2018 – September 2021**

|  | **Responders** | |  | **Non-responders** | |
| --- | --- | --- | --- | --- | --- |
| **Age (years)** | **n** | **%** |  | **n** | **%** |
| Overall |  |  |  |  |  |
| 62-66 | 364 | 17.4 |  | 511 | 9.1 |
| 67-71 | 514 | 24.5 |  | 867 | 15.4 |
| 72-76 | 427 | 20.4 |  | 802 | 14.2 |
| 77-81 | 447 | 21.3 |  | 1177 | 20.8 |
| 82-86 | 253 | 12.1 |  | 1151 | 20.4 |
| 87-91 | 90 | 4.3 |  | 1141 | 20.2 |
| Total | 2095 |  |  | 5649 |  |
| Men |  |  |  |  |  |
| 62-66 | 169 | 16.3 |  | 265 | 10.2 |
| 67-71 | 253 | 24.5 |  | 407 | 15.7 |
| 72-76 | 209 | 20.2 |  | 364 | 14.0 |
| 77-81 | 231 | 22.3 |  | 538 | 20.7 |
| 82-86 | 129 | 12.5 |  | 531 | 20.4 |
| 87-91 | 43 | 4.2 |  | 495 | 19.0 |
| Total | 1034 |  |  | 2600 |  |
| Women |  |  |  |  |  |
| 62-66 | 195 | 18.4 |  | 246 | 8.1 |
| 67-71 | 261 | 24.6 |  | 460 | 15.1 |
| 72-76 | 218 | 20.5 |  | 438 | 14.4 |
| 77-81 | 216 | 20.4 |  | 639 | 21.0 |
| 82-86 | 124 | 11.7 |  | 620 | 20.3 |
| 87-91 | 47 | 4.4 |  | 646 | 21.2 |
| Total | 1061 |  |  | 3049 |  |

Percentages are column percentages; non-responders older than 91 years (n=129) were excluded.

**Suppl. Table 2 Assumed quantitative bias parameters for the bias analysis of potential non-response**

|  | **Dry eye prevalence among responders** | **Assumed prevalence scenarios**  **among non-responders in percent of the prevalence of the responders** | | | |
| --- | --- | --- | --- | --- | --- |
|  |  | **Nondifferential non-response bias in relation to age** | | **Differential non-response bias in relation to age** | |
|  | **Prev (%)** | **#1** | **#2** | **#3** | **#4** |
| Men |  |  |  |  |  |
| 62-66 | 9.7 | 110% | 120% | 105% | 110% |
| 67-71 | 21.5 | 110% | 120% | 110% | 115% |
| 72-76 | 20.9 | 110% | 120% | 115% | 120% |
| 77-81 | 22.1 | 110% | 120% | 120% | 125% |
| 82-86 | 29.9 | 110% | 120% | 125% | 130% |
| 87-91 | 17.9 | 110% | 120% | 130% | 135% |
| Women |  |  |  |  |  |
| 62-66 | 35.6 | 110% | 120% | 105% | 110% |
| 67-71 | 42.3 | 110% | 120% | 110% | 115% |
| 72-76 | 41.8 | 110% | 120% | 115% | 120% |
| 77-81 | 44.0 | 110% | 120% | 120% | 125% |
| 82-86 | 51.8 | 110% | 120% | 125% | 130% |
| 87-91 | 40.5 | 110% | 120% | 130% | 135% |

Legend: the meaning is illustrated for women aged 87-91 years and bias scenario #2: the prevalence of dry eye syndrome (DES) among the responders is 40.5%; the assumed prevalence among nonresponders is 120% of the prevalence of the responders, therefore 40.5 * 1.20 = 48.6%; the overall (responders + nonresponders) prevalence of DES is calculated by adding DES cases and total number of subjects from responders and nonresponders

**Suppl. Table 3 Overall and sex-specific prevalence of dry eye syndrome in relation to the non-response bias scenarios, Heinz Nixdorf Recall study, Germany, January 2018 – September 2021**

|  | **Overall** | **Men** | | **Women** |
| --- | --- | --- | --- | --- |
| DES Prevalence among responders | 31.5 | 20.4 | | 42.2 |
|  |  |  | |  |
| DES Prevalence after including non- responders | | |  |  |
| Bias scenario |  |  | |  |
| #1 | 35.4 | 22.7 | | 46.5 |
| #2 | 37.8 | 24.2 | | 49.8 |
| #3 | 37.8 | 24.2 | | 49.8 |
| #4 | 39.0 | 25.0 | | 51.4 |

**Suppl. Fig 1 Absolute number of participants on the eye survey by sex and age among 2095 men and women of the Heinz Nixdorf Recall study, Germany, January 2018 – September 2021**

| 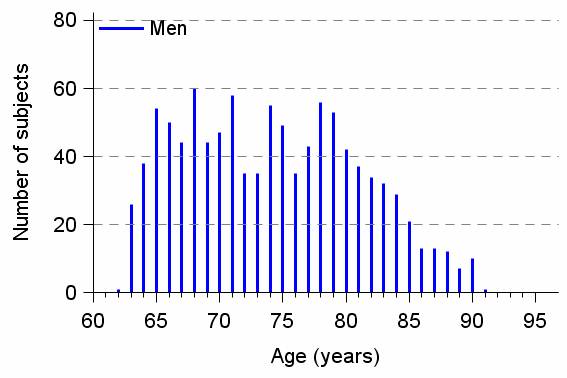 | 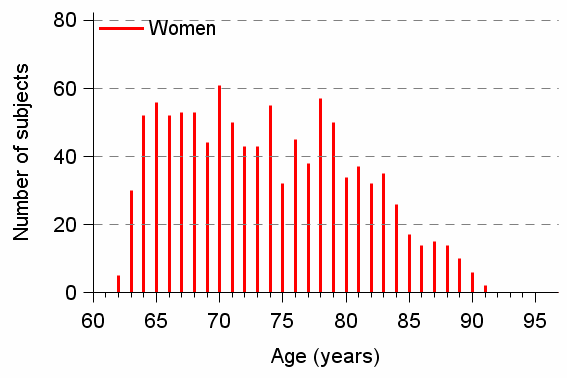 |
| --- | --- |

**Suppl. Fig. 2 Sex- and age-specific prevalence of dry eye syndrome in relation to varying quantitative non-response bias parameters**

| 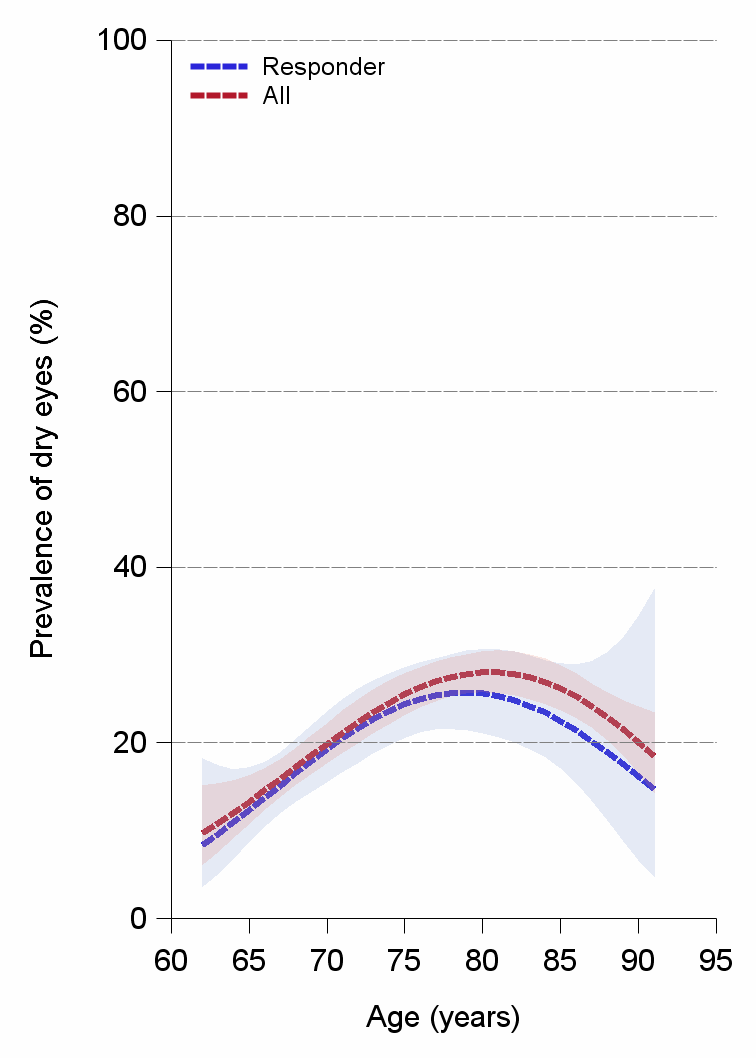 | 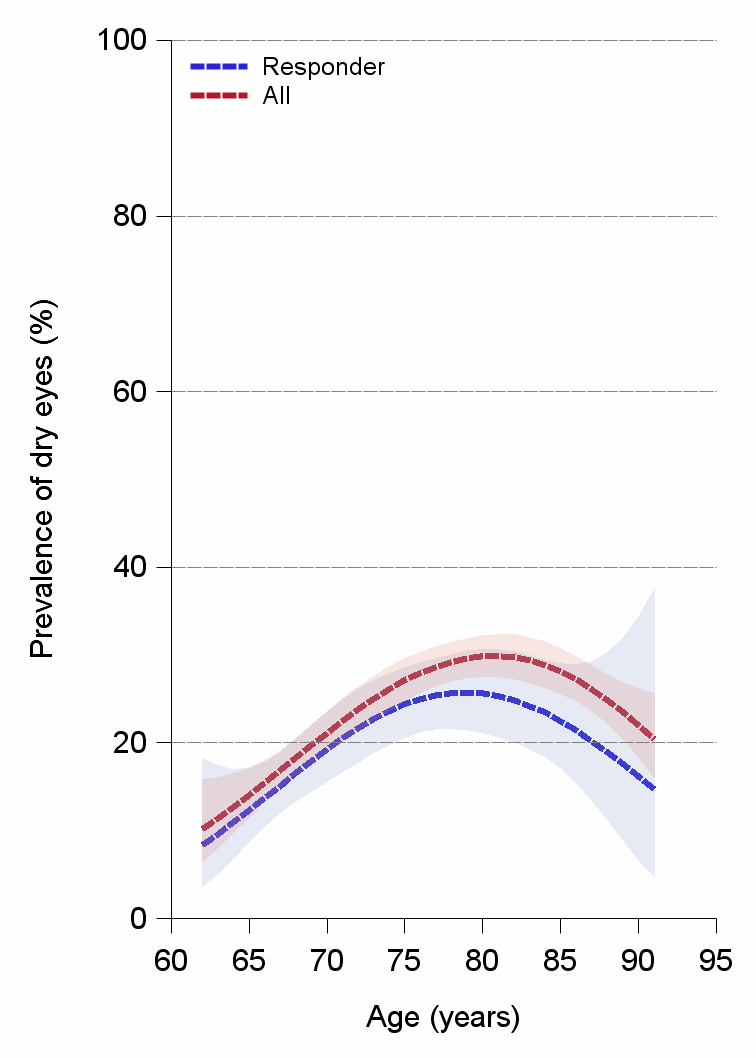 | 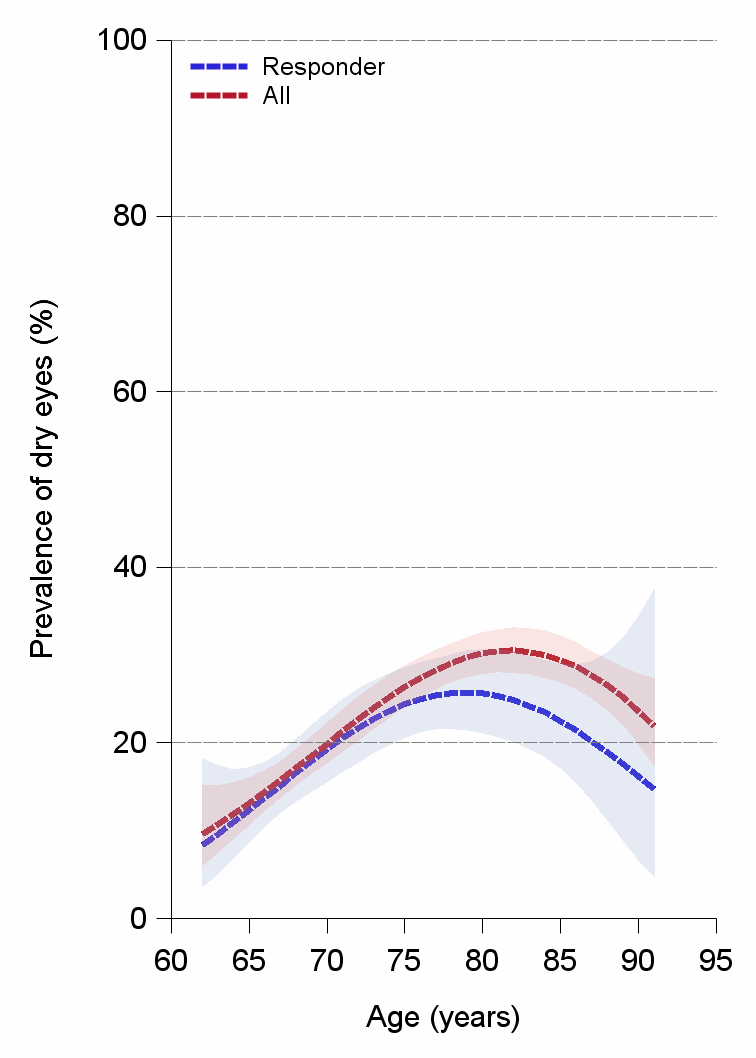 | 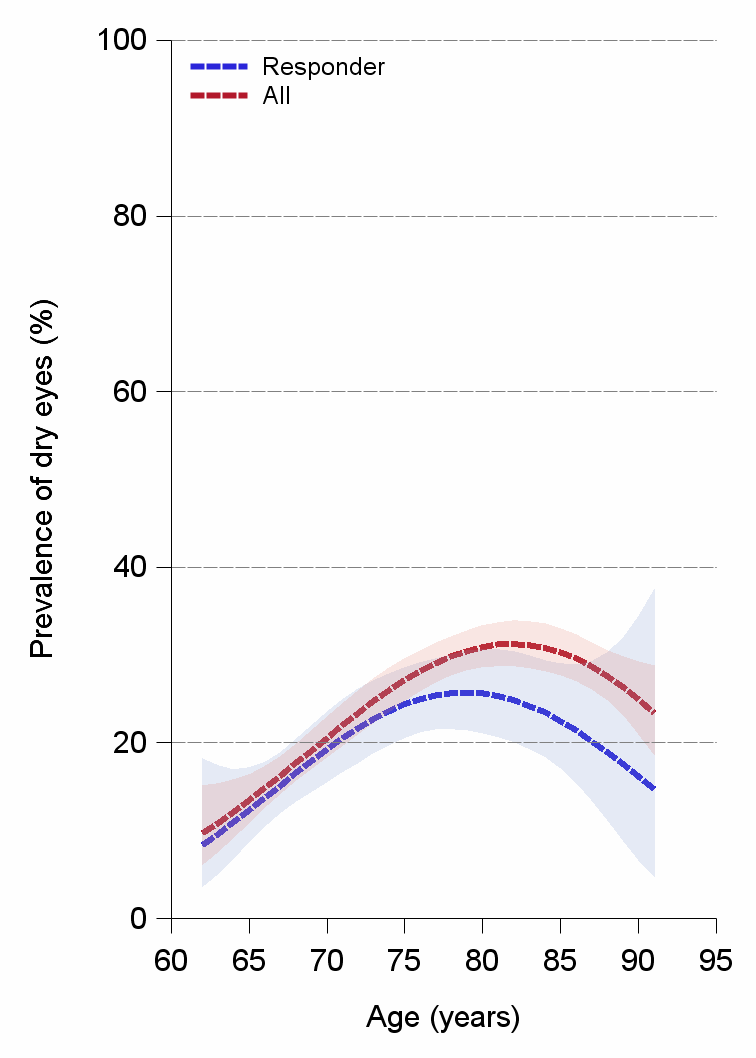 |
| --- | --- | --- | --- |
| Scenario #1 (men) | Scenario #2 (men) | Scenario #3 (men) | Scenario #4 (men) |
| 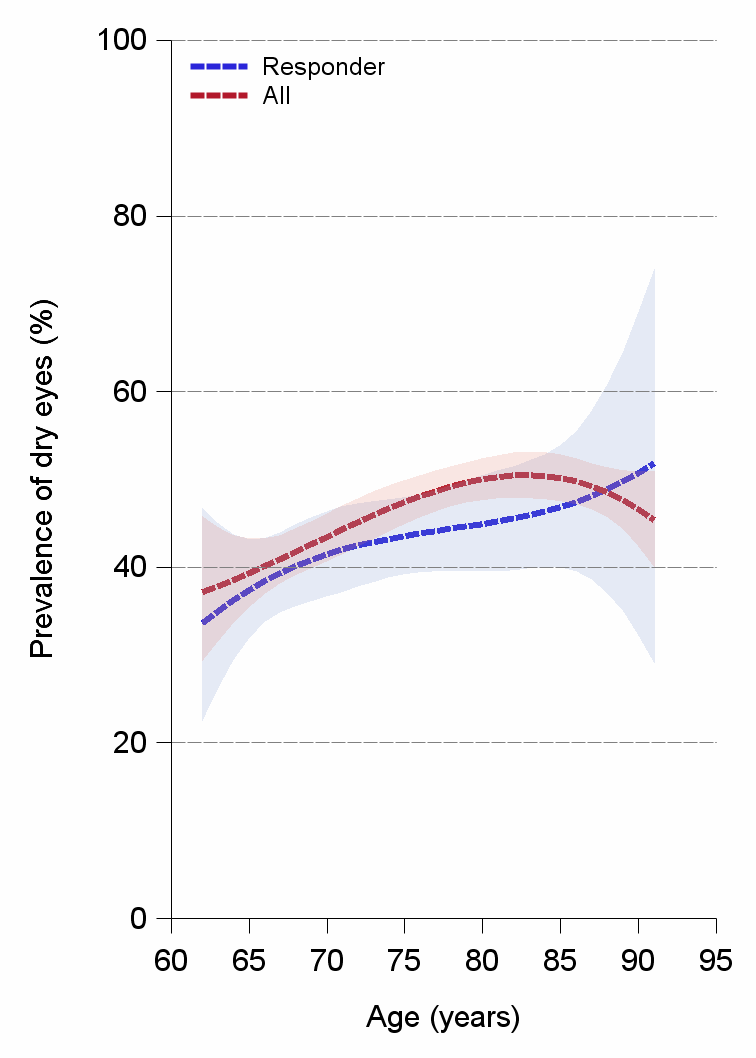 | 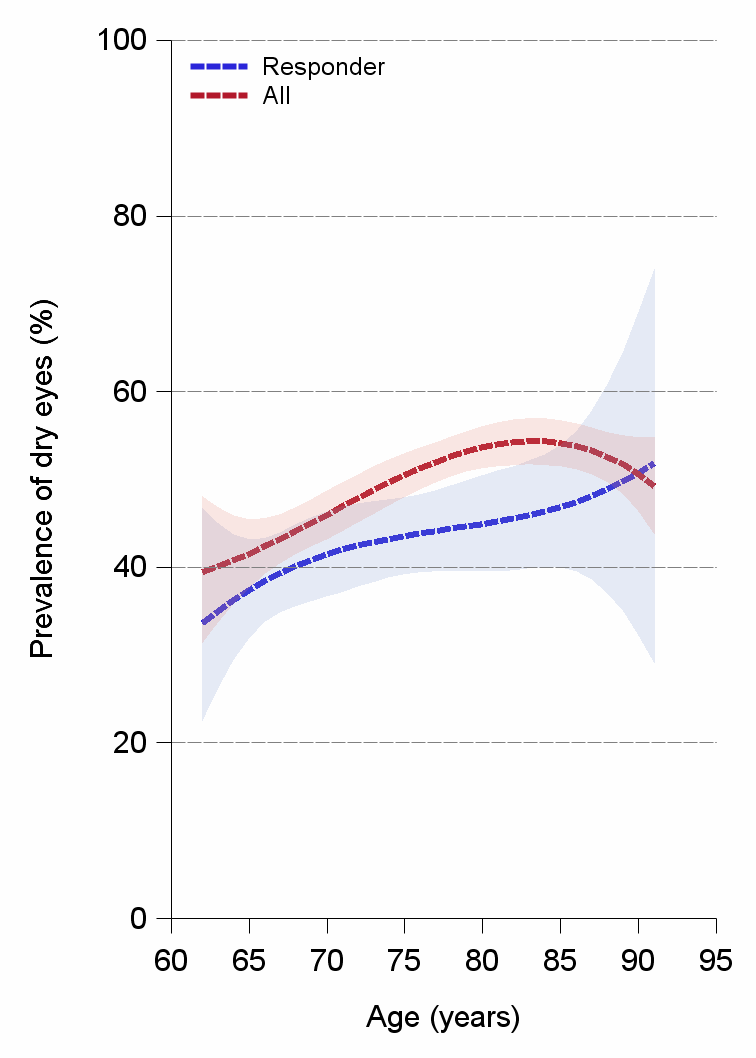 | 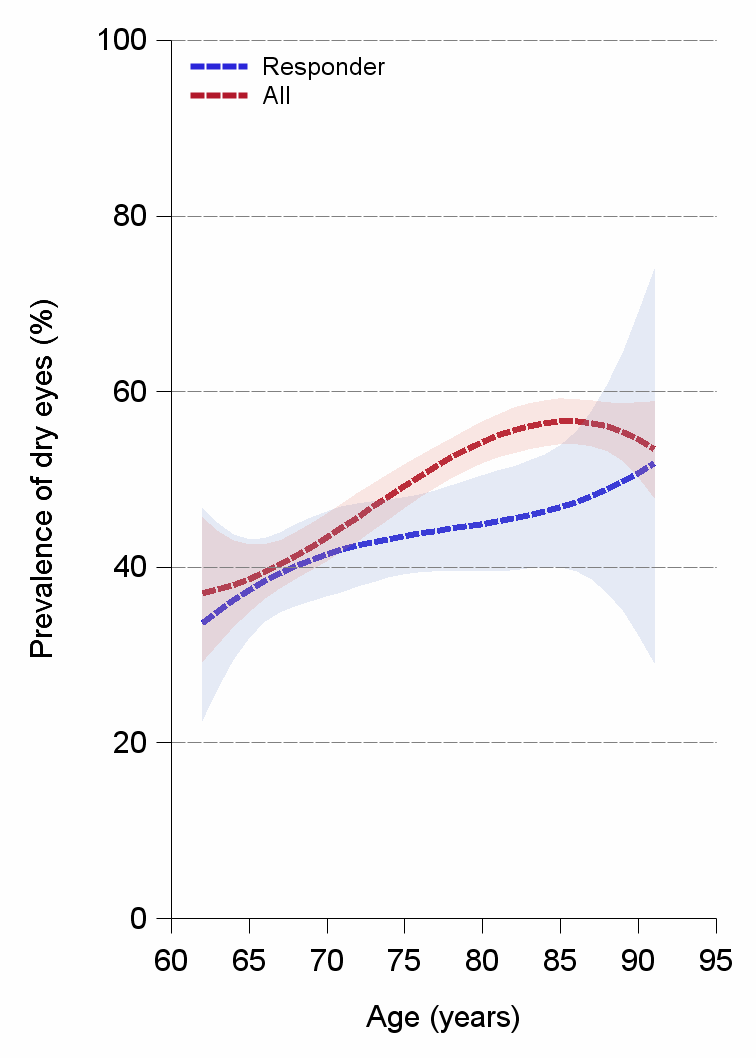 | 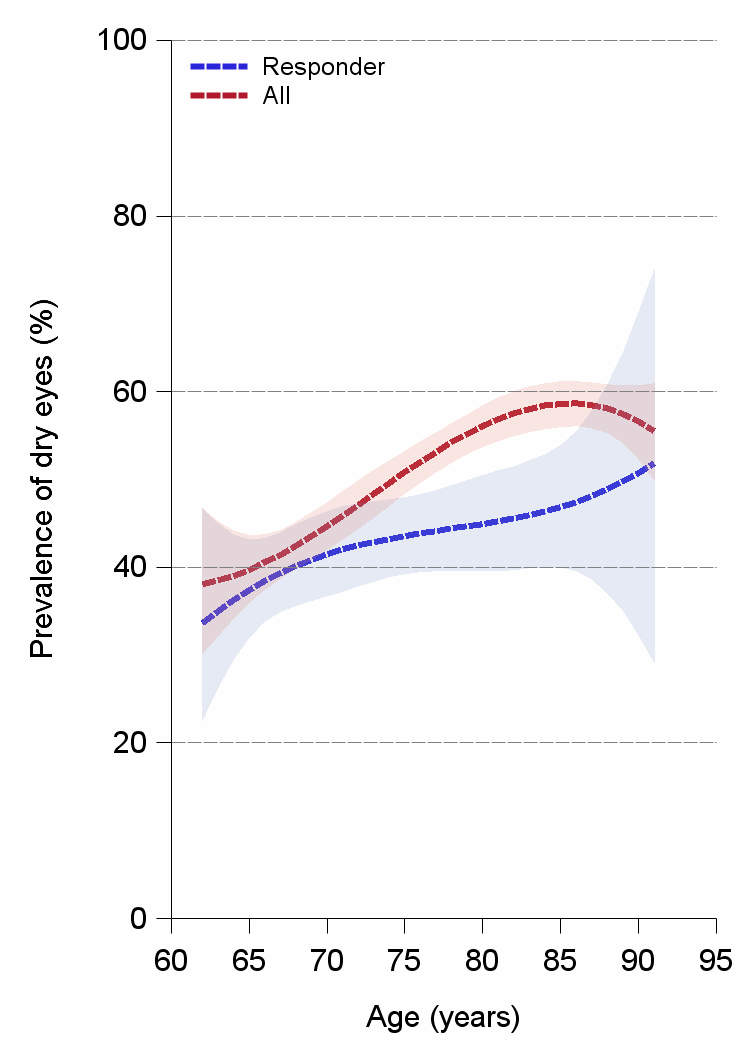 |
| Scenario #1 (women) | Scenario #2 (women) | Scenario #3 (women) | Scenario #4 (women) |

Flexible logistic regression modelling included age, age^2^, and age^3^

**Supplementary Figure 3 Recruitment results at baseline (2000-2003) and in the follow-up (2018-2021), in which the DES survey took place, including a presentation of non-responders, Heinz Nixdorf Recall Study, Germany**


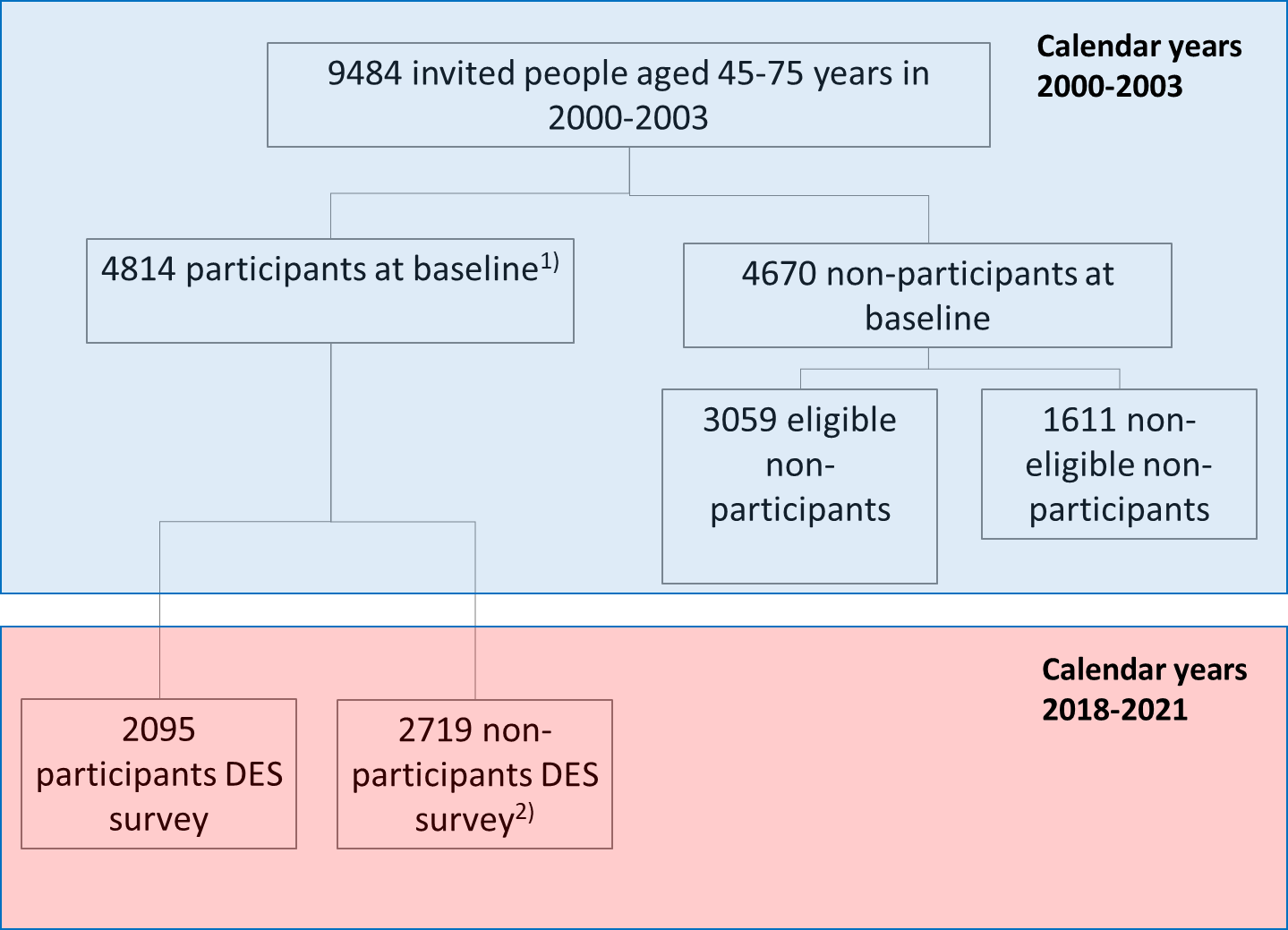


Legend Suppl. Figure 3

1) includes n=327 subjects with manifest coronary artery disease at baseline; 2) includes 1132 deceased subjects, 812 subjects who refused and 775 subjects who did not answer the postal questionnaire. For the quantitative bias analysis on potential non-response, subjects aged < 92 years among the 2719 nonparticipants of the DES survey and among the 3059 nonparticipants at baseline were used to estimate the bias-corrected prevalence of DES
