# Supplementary material for: Synergism between coexisting eye diseases and sex in increasing the prevalence of the dry eye syndrome
Source: Sci Rep. 2024 Jan 3;14:314. doi: 10.1038/s41598-023-50871-1 (PMC10764946; doi:10.1038/s41598-023-50871-1)
Supplement: Supplementary file 2 — Supplementary Information 2. [file 41598_2023_50871_MOESM2_ESM.docx]

**Dry-Eye Questionnaire (Women’s Health Study, WHS)**

1. Have you ever been diagnosed with dry eye by a medical doctor?

(A) Yes

(B) No

2. How often do your eyes feel dry (not moist enough)?

(A) Constantly

(B) Often

(C) Sometimes

(D) Never

3. How often do your eyes feel irritated?

(A) Constantly

(B) Often

(C) Sometimes

(D) Never

4. Have you in the last 12 months been treated for dry eye, and if so by whom? (Multiple answers possible.)

(A) No.

(B) Yes, by an opthalmologist.

(C) Yes, by a medical doctor other than an opthalmologist.

(D) Yes, by a pharmacist or other non-medical specialist.

(E) Yes, by myself.

**Additional question on eye diseases [designed by the authors Stang et al.]**

Have you ever been to an ophthalmologist?

No

Yes

If yes, please name the opthalmologist who last treated you.

Physician/hospital ___________________________

Date (month, year) ___________________________

Have you ever been diagnosed with (German: “grauer Star” or) cataracts by a doctor?

No

Yes

If yes, In which year was a cataract diagnosed for the first time?

Year __________

Have you received medical treatment by a doctor for (German “grauer Star” or) cataracts in the last 12 months?

No

Yes

Have you ever been diagnosed with (German: “grüner Star” or) glaucoma by a doctor?

No

Yes

If yes, In which year was a (German: “grüner Star” or) glaucoma diagnosed for the first time?

Year __________

Have you received medical treatment by a doctor for (German “grüner Star” or) glaucoma in the last 12 months?

No

Yes

Have you ever been diagnosed with macula degeneration by a doctor?

No

Yes

If yes, In which year was a macula degeneration diagnosed for the first time?

Year __________

Have you received medical treatment by a doctor for macula degeneration in the last 12 months?

No

Yes
